# Predictive genetic testing for BRCA1/2 in a UK clinical cohort: three-year follow-up

**DOI:** 10.1038/sj.bjc.6603610

**Published:** 2007-02-06

**Authors:** C Foster, M Watson, R Eeles, D Eccles, S Ashley, R Davidson, J Mackay, P J Morrison, P Hopwood, D G R Evans, and Psychosocial Study Collaborators

**Affiliations:** 1 Macmillan Research Unit, School of Nursing and Midwifery, University of Southampton, Southampton SO17 1BJ, UK; 2 Psychology Research Group, The Institute of Cancer Research, Sutton SM2 5NG, UK; 3 Translational Cancer Genetics Team & Cancer Genetics Unit, Royal Marsden NHS Trust, London & Sutton SM2 5PT, UK; 4 Wessex Clinical Genetics Service, Princess Ann Hospital, Southampton SO16 5YA, UK; 5 Department of Computing, Royal Marsden NHS Trust, Sutton SM2 5PT, UK; 6 Institute of Medical Genetics, Yorkhill NHS Trust, Glasgow G3 8SJ, UK; 7 Genetics Centre, Institute of Child Health, London WC1N, UK; 8 Medical Genetics, Belfast City Hospital, Belfast BT9 7AB, UK; 9 Christie Hospital, Manchester M20 4BX, UK; 10 Department of Medical Genetics, St Mary's Hospital, Manchester M13 0JH, UK

**Keywords:** genetic testing, risk management, psychological; BRCA1/2

## Abstract

This prospective multicentre study assesses long-term impact of genetic testing for breast/ovarian cancer predisposition in a clinical cohort. Areas evaluated include risk management, distress and insurance problems 3 years post-testing. Participants are adults unaffected with cancer from families with a known BRCA1/2 mutation. One hundred and ninety-three out of 285 (70% response) participants at nine UK clinical genetics centres completed assessments at 3 years: 80% female; 37% carriers of a BRCA1/2 mutation. In the 3 years, post-genetic testing carriers reported more risk management activities than non-carriers. Fifty-five per cent of female carriers opted for risk reducing surgery; 43% oophorectomy; and 34% mastectomy. Eighty-nine per cent had mammograms compared with 47% non-carriers. Thirty-six per cent non-carriers ⩾50 years did not have a mammogram post-test. Twenty-two per cent male carriers had colorectal and 44% prostate screening compared with 5 and 19% non-carriers respectively. Seven per cent carriers and 1% non-carriers developed cancer. Distress levels did not differ in carriers and non-carriers at 3-year follow-up. Forty per cent of female carriers reported difficulties with life and/or health insurance. Given the return to pre-test levels of concern among female non-carriers at 3 years and a substantial minority not engaging in recommended screening, there appears to be a need to help some women understand the meaning of their genetic status.

A BRCA1 or BRCA2 gene mutation is associated with increased risk of breast and/or ovarian cancer (Miki *et al*, 1994; Wooster *et al*, 1995). This presents a number of challenges for the medical community, patients and their families. Female carriers of BRCA1/2 mutations (hereafter referred to as female carriers) are at substantially increased risk of developing breast and/or ovarian cancer (Ford *et al*, 1998). Male carriers of BRCA1/2 mutations (hereafter referred to as male carriers) have an increased risk of prostate (substantial for BRCA2) and bowel cancer (Ford *et al*, 1994). Male carriers of BRCA2 mutations are at risk of developing breast cancer (Easton *et al*, 1997). Genetic testing is becoming more widely available and evaluation of the long-term psychological impact and risk management strategies used are required. There are few reports on long-term consequences of BRCA1/2 testing beyond 1 year and most research focuses on women. This is the first paper to report uptake of risk management options, psychological distress and insurance problems in a large UK clinical cohort of men and women 3 years following predictive testing for BRCA1/2 mutations.

## 

### Risk management

Individuals at increased cancer risk are usually offered regular screening for early detection and/or risk reducing surgery. In the year following genetic testing, female carriers receive more cancer screening and risk-reducing surgery relative to pre-test levels and non-carriers (Watson *et al*, 2004). In our UK cohort, 28% of carriers had bilateral risk reducing mastectomy (BRRM) and 31% oophorectomy (BRRO) in the year following predictive testing. In the USA, women are less likely to have BRRM (0–15%) than BRRO (13–51%) (Lerman *et al*, 2000; Scheuer *et al*, 2002; Botkin *et al*, 2003; Schwartz *et al*, 2003) whereas in the Netherlands the BRRM/BRRO rate is around 50% for both (Meijers-Heijboer *et al*, 2000; Lodder *et al*, 2002).

Assessment of risk management beyond the first year is limited and does not include data from men. One study with long-term follow-up (5 years post-testing) (van Oostrom *et al*, 2003) found most carriers (19 out of 23) had BRRM with fewer (12 out of 23) having BRRO. Other forms of risk management were not described. Genetic testing may be of limited value if recommended screening strategies are not maintained over time. Longer term follow-up will indicate whether individuals are receiving appropriate screening and highlight areas for concern.

Female carriers are typically referred to their local screening service or breast/gynaecological surgeons for management of their risk. However, in the year following genetic testing some UK health services have appeared slow to respond (Watson *et al*, 2004). Guidelines have since been published for the management of risk associated with familial breast cancer within the UK National Health Service (NHS: provides clinical services free to all at the point of delivery) (McIntosh *et al*, 2004). There are no UK guidelines for the management of ovarian cancer risk or risk management for male carriers, although PSA screening is likely to be offered. We assess risk management in the 3 years following BRCA1/2 mutation testing for breast and ovarian cancer risk in women and prostate and colorectal cancer risk in men.

### Psychological distress

Research has focused on psychological distress experienced by women following predictive BRCA1/2 testing (Lodder *et al*, 2001a; Meiser *et al*, 2002; Schwartz *et al*, 2002; van Oostrom *et al*, 2003). There is evidence of short-term (a few months following the test result) adverse effects on emotional well-being with female carriers experiencing an increase in cancer worry (Meiser *et al*, 2002; Watson *et al*, 2004). Women not expecting to be a carrier (Schwartz *et al*, 2002) or experiencing high levels of distress before genetic testing (Lodder *et al*, 2001a) are most vulnerable to distress later. The longer term impact is less clear. Distress has been shown to return to pre-test levels a year after genetic testing in this clinical UK cohort (Watson *et al*, 2004). However, in a smaller Australian cohort distress remained elevated among carriers a year following testing (Meiser *et al*, 2002). Non-carriers on the other hand are consistently reported to experience reduced levels of cancer worry following testing, which is maintained at 1 year (Meiser *et al*, 2002; Watson *et al*, 2004).

Far less is known about concerns in the longer term. Increases in generalised anxiety and depression (although not to clinically significant levels) have been reported up to 5 years post-test result in a small group of female carriers (*N*=23) and non-carriers (*N*=42) although cancer-related concern did not increase over this period (van Oostrom *et al*, 2003). Men have rarely been included in studies assessing the impact of BRCA1/2 genetic testing (Lodder *et al*, 2001b). We assess distress in this study to identify psychological sequelae for women and men following predictive genetic testing for BRCA1/2.

### Insurance

There has been considerable debate about possible reactions of the insurance industry to genetic testing. Concern has been expressed about insurance discrimination (Morrison *et al*, 1999, 2000). There is currently a voluntary moratorium in the UK, recently extended to November 2011 (subject to review in 2008), on the use of predictive genetic test results by insurance companies when calculating premiums for life insurance policies under £500 000, critical illness and income protection under £300 000 per policy (Morrison, 2005). In the year following BRCA1/2 testing, 20% of women with a BRCA1/2 mutation reported problems with insurance (Watson *et al*, 2004). We assess the level of insurance problems in the 3 years post-testing to clarify whether this is a continuing problem.

In summary, this study includes men and women, unaffected by cancer at the time of genetic testing, attending nine genetic centres undertaking the majority of BRCA1/BRCA2 testing in clinical settings across the UK. Risk management, distress and insurance problems 3 years post-genetic testing are documented along with patient reported cancer rates and clinical interventions (e.g., biopsies, surgical procedures, screening). Three key questions are addressed. In the 3 years post-testing:

1. How does BRCA1/2 testing impact upon risk management?
2. What levels of psychological distress exist?
3. What level of insurance problems is reported?

## MATERIALS AND METHODS

### Participants

A total of 285 adults recruited from nine UK centres were followed up for 3 years after genetic testing. Eligible participants were unaffected by cancer and from families with a BRCA1/2 mutation identified in an affected blood relative. Participants had a 50% (lower if an intervening relative had died) risk of inheriting a BRCA1/2 mutation.

### Procedure

Using a prospective design, participants were recruited from genetics clinics between 1997 and 2000. Participants completed a baseline (pre-genetic test) questionnaire and 3-year follow-up assessment. Trent multicentre research ethics committee and all local research ethics committees approved the study. Written consent was obtained at both time points.

### Measures

Demographic data were collected at baseline: age, education level, marital status, number of children and employment status. BRCA1/2 mutation status was collected from clinic records at the end of the study. Individuals found to carry a BRCA1/2 mutation are referred to as ‘carriers’ and those who do not ‘non-carriers’. Data on cancer rates were collected from participants.

#### Risk management

Women reported risk management undertaken in the 3 years following their genetic test using a checklist; mammography, BRRM (removal of healthy breasts to reduce risk), BRRO (removal of healthy ovaries to reduce risk), ovarian ultrasound (Ov US), tamoxifen, clinical examination of breasts by a doctor (CBE) and breast self-examination (BrSE). Frequency of BrSE and breast/ovarian biopsy rates were recorded. These data were compared with women's responses at baseline indicating whether they had already undergone these procedures. Men reported colorectal, prostate or other cancer screening following genetic testing.

#### Mental health and cancer related concerns:

*General health questionnaire 28 – GHQ28* (Goldberg and Hillier, 1979): this 28-item measure assesses psychiatric disorder (cases) in non-psychiatric populations and has been used with medical patients. A total score on the GHQ28 (binary scoring) ranges from 0 to 28. A cutoff score of ⩾5 is recommended by the test authors as being clinically significant, that is a score ⩾5 indicates psychiatric disorder. However, Hopwood *et al* (1998) recommend ⩾10 (binary scoring) as the cutoff for women with familial cancer risk to reduce overestimation of cases and this was used in the present study. Symptoms were assessed in male and female participants.

*Cancer worry scale-revised – CWS-R* (Lerman *et al*, 1993; Watson *et al*, 1999; Foster *et al*, 2002): this 6-item scale assesses degree of worry about developing cancer over the previous 7 days. A total score on the CWS-R ranges from 6 to 24. A high score indicates greater worry about cancer. No clinical cutoffs are currently available. The follow-up data yielded an alpha coefficient of 0.87 (Watson *et al*, 2004). Cancer worry was assessed in male and female participants.

*Insurance difficulties*: Women indicated problems with life, health or disability insurance post-genetic test and were invited to specify the nature of these problems. They were not asked if they had declared the test result to their insurer.

### Statistical method

The association between categorical variables was examined using Fisher's exact test or *χ*^2^ with Yates correction where appropriate. For ordered categorical variables Mann–Whitney (MW) test for trend was used. Age was analysed as a continuous variable and participants were divided into three age groups (<35; 35–49; >50) to reflect variations that might occur in risk management especially in relation to mammography. Women under 35 years are unlikely to receive a mammogram and women over 50 years of age receive regular mammograms as part of the UK National Screening Programme. Where the <35 and 35–49 year age groups have similar reports compared to the ⩾50s, the two younger groups are reported as one (<50).

Scores from GHQ28 and CWS-R were treated as continuous variables. Normality was tested using the Kolmorgorov–Smirnov statistic and parametric or non-parametric statistics used as appropriate. Scores are summarised using mean (m) and standard deviation (s.d.) or median and range. Groups were compared using analysis of variance or Kruskal–Wallis (KW) test. Matched data were used to compare individuals that completed both baseline and 3-year follow-up questionnaires. Associations between scores are summarised by Pearson or Spearman correlation coefficients. Changes in frequency of BrSE post-testing were explored separately for carriers and non-carriers by *χ*^2^ and KW tests. Predictors of cancer worry were investigated by MW.

## RESULTS

Two hundred and eighty-five individuals recruited at baseline received a genetic test result: 100 (35%) carriers and 185 (65%) non-carriers. At 3-year follow-up 277 questionnaires were sent out. Eight individuals were not sent a follow-up questionnaire: two participants had moved house, one man had died of cancer (carrier), three participants were persistent non-responders during one year follow-up and two had previously withdrawn from the study. Twelve individuals (4%) actively declined participation by returning blank questionnaires (as requested). One hundred and ninety-three (70%) participants returned follow-up questionnaires. A similar proportion of carriers and male participants responded at baseline and follow-up. More responders were married than non-responders (*P*=0.02; *χ*^2^). There were no other significant differences.

Socio-demographic characteristics of the participants are presented in Table 1. Sixty per cent of women and 54% of men are non-carriers. Of the 71 carriers, 48 (68%) had BRCA1, 22 (31%) BRCA2 and 1 (1%) both BRCA1 and BRCA2 mutations. Of carriers, 48 women and 10 men were <50; five women and eight men ⩾50. Eighty-four per cent described themselves as Caucasian. Most men (74%) and women (66%) were employed. Most men (90%) and women (86%) were married or cohabiting. Forty-nine per cent of men and 36% of women reported receiving higher education. Five (7%) carriers and one (1%) non-carrier reported a cancer diagnosis since genetic testing: two ovarian and one breast cancer (carriers), two prostate (one carrier; one elderly non-carrier) and one skin cancer (carrier).

Participants at each centre were compared on demographic variables. Three centres accounted for 83% of participants. There were no differences across the three larger centres. Participants (*n*=33) from the six smaller centres were younger (*P*=0.04; KW).

### How does BRCA1/2 genetic testing impact upon risk management in the long term?

#### Female participants

Table 2 illustrates risk management options undertaken at baseline and those undertaken in the 3 years post-testing according to carrier status and age. There were no differences in risk management options between carriers and non-carriers at baseline. This was not the case 3 years later. Mammography rates were significantly higher (*P*<0.001; *χ*^2^) in carriers (89%) compared to non-carriers (46%). Thirty-six per cent of non-carriers <50 years and 65% of non-carriers ⩾50 years had a mammogram post-genetic test. The majority of carriers and non-carriers reported performing BrSE but carriers were more likely to report increased frequency of BrSE than non-carriers (*P*=0.04; MW_trend_).

One non-carrier had BRRM pre-test (not BRRO), four carriers and 10 non-carriers (9%) had BRRO pre-test. Three per cent (*N*=3) non-carriers had their ovaries removed as part of a hysterectomy following testing. Fifty-four per cent (21 out of 39) of carriers with children had BRRO compared to none (0 out of 10) without (*P*<0.0001; *χ*^2^). Age was not associated with BRRO in carriers (*P*=0.3, MW_trend_). Of the carriers opting for BRRM all were <50 and 11 were <40 at baseline (1 was <30). Ten carriers had both BRRM and BRRO post-genetic test. Overall, 27 out of 41 (66%) carriers with children had risk reducing surgery compared to 1 out of 10 without children (*P*=0.01; Fisher). Significantly more carriers had Ov US (*P*<0.001; *χ*^2^) and biopsies (*P*=0.05; *χ*^2^) compared to non-carriers. Risk management options undertaken by BRCA1 and BRCA2 carriers did not differ significantly; 17 out of 35 (49%) BRCA1 carriers had BRRO by 3 years compared to four out of 14 (29%) BRCA2 carriers (*P*=0.3; Fisher).

BRRM rates for carriers varied from centre to centre, 8–55%. One centre had a significantly higher rate of BRRM than others (*P*=0.02; Fisher). Baseline cancer worry was not a significant predictor of risk reducing surgery by 3 years (*P*=0.4; KW) and risk reducing surgery did not reduce cancer worry (*P*=0.8; KW) in carriers by 3-year follow-up. However, numbers are small and this effect should be interpreted with caution as it is likely to be underpowered. There was no evidence that cancer worry at baseline influenced uptake of other risk management options. Since almost all women reported practicing BrSE, a relationship with cancer worry can not be tested.

### Male participants

Four out of 18 (22%) male carriers had screening for colorectal and eight out of 18 (44%) for prostate cancer post-genetic test. This compares to one out of 21 (5%) non-carriers screened for colorectal and four out of 21 (19%) for prostate cancer. One additional non-carrier underwent a non-specified biopsy. Of the carriers, the median age of the men who had prostate screening was 54 years compared to 37 years for those not screened. Three out of eight (38%) carriers ⩾50 years had not received screening post-genetic testing. There was no relationship between cancer worry at 3 years and self-reported risk management; however, these numbers are too small to give an adequately powered analysis.

### What levels of psychological distress exist in the long term?

At 3 years more women than men were identified as cases using the GHQ28: 18% (nine out of 51) female carriers and 17% (16 out of 95) non-carriers compared to 11% (two out of 18) of male carriers and 10% (two out of 21) non-carriers. There were no significant changes in caseness over time. There was no difference at baseline (men: *P*=0.4; women: *P*=1.0; KW) or 3 years (men: *P*=0.8; women *P*=0.7; KW) in general mental health between carriers and non-carriers. However, female carriers did report higher generalised distress scores at 3 years than at baseline (*P*=0.03) (Table 3).

There was no difference in cancer worry at baseline (women: *P*=0.6; KW) or 3 years (men: *P*=0.9; women: *P*=0.2; KW) when comparing carriers and non-carriers. At baseline younger women (<50 years) were more worried about developing cancer than older women (*P*<0.001; KW). There was no such difference at 3 years between older and younger women (carriers: *P*=0.5; non-carriers: *P*=1.0; KW) or men (carriers: *P*=0.8; non-carriers: *P*=0.7; KW). Female carriers were less worried at 3 years than they reported at baseline (*P*=0.03).

### What level of insurance problems is reported?

Overall, 21 (40%) carriers reported insurance problems; three had not applied for insurance and two did not answer the question (10% in total). In total, 10 (19%) carriers and four (4%) non-carriers reported difficulties obtaining life insurance (*P*=0.006; Fisher). Six (11%) carriers and 2 (2%) non-carriers had difficulties obtaining health insurance. One (2%) carrier reported problems with disability insurance. Six (11%) carriers and one (1%) non-carrier reported an increased premium (*P*=0.008; Fisher). The non-carrier's premium was reduced after the test result. Although invited to do so few women provided details of problems experienced.

## DISCUSSION

The study participants represent a significant proportion of patients undergoing predictive genetic testing between 1997 and 2000. This is the first report of data three years following genetic testing for BRCA1/2 in a clinical cohort in the UK. These data indicate areas where there may be a need for intervention to inform and reassure people tested in the longer term. While carriers engaged in more screening and surgical risk management there are some potential areas of concern.

Over a third of non-carriers ⩾50 years had not had a mammogram in the 3 years following their test despite the UK national breast screening guidelines. We are currently investigating whether this represents a failure to offer mammography or lack of attendance in this group of non-carriers thinking they are now exempt from breast cancer risk. While the uptake of mammography in the National Breast Screening Programme is around 75% (Department of Health, 2004), some women in this study may be deriving false reassurance from their non-carrier result. Botkin *et al* (2003) did not find evidence that non-carriers were falsely reassured 2 years post-test result in their study.

Over a third of non-carriers <50 years reported having a mammogram post-genetic test. However, the National Breast Screening Programme does not screen population risk women until 50 years. While some of these women will have turned 50 in the 3 years since testing, some may be reluctant to relinquish screening that was in place before testing. Some women may continue screening due to other suspected mutations in the family; however, there appears to be some inappropriate screening within the non-carrier group.

Female carriers reported higher generalised distress 3 years following testing than they did at baseline. This was also found by van Oostrom *et al* (2003) in their small cohort of female carriers and non-carriers who reported higher levels of generalised anxiety and distress 1–5 years following the test result (although not clinically significant). The increase in generalised distress in the carrier group suggests that some women may benefit from additional information or support in the longer term although this may not be related to concern about developing cancer.

Female non-carriers did not report significantly different levels of cancer worry compared to baseline. However, in the year following predictive testing, female non-carriers reported a substantial reduction in cancer worry, which is not maintained in the long term (Watson *et al*, 2004). Mean scores give a general picture and the range of scores 3 years following genetic testing indicate that some non-carriers have higher scores which is of potential concern. Further investigation of both carriers and non-carriers is warranted to clarify the particular support needs that may not currently be met in the longer term.

Our findings demonstrate that carriers are more likely than non-carriers to engage in risk management strategies post-genetic test where no differences existed at baseline.

Overall, most female carriers (55%) opted for risk reducing surgery following their genetic test. Our data indicate that most women who opted for BRRM did so in the year following BRCA1/2 testing while some women waited longer for BRRO. The number of carriers having BRRO (43%) in the 3 years post-genetic testing was higher than 1 year post-testing in the same cohort (Watson *et al*, 2004). This figure is comparable to rates 1 year post-test reported in the USA (Lerman *et al*, 2000; Scheuer *et al*, 2002; Botkin *et al*, 2003; Schwartz *et al*, 2003) and slightly lower than reports from the Netherlands 1 (Meijers-Heijboer *et al*, 2000; Lodder *et al*, 2002) and 5 years following testing (van Oostrom *et al*, 2003). For BRRM (34%) the rate is similar to the rate reported at 1 year in this cohort (Watson *et al*, 2004). The BRRM rate is higher than in the USA (Lerman *et al*, 2000; Scheuer *et al*, 2002; Botkin *et al*, 2003; Schwartz *et al*, 2003) and lower than in the Netherlands at 1–2 years (Meijers-Heijboer *et al*, 2000; Lodder *et al*, 2002) and 5 years post-testing (van Oostrom *et al*, 2003).

UK guidelines regarding risk management for women with familial breast cancer have recently been published (McIntosh *et al*, 2004). These National Institute for Clinical Excellence (NICE) guidelines indicate that BRRM and/or BRRO are appropriate for BRCA1/2 carriers and should be managed by a multidisciplinary team. There are no guidelines for the management of women at risk of ovarian cancer, although research in this area is underway (UKFOCSS: UK Familial Ovarian Cancer Screening Study). Female carriers of a BRCA1/2 mutation may be offered regular trans-vaginal ultrasound and, where appropriate, CA125 testing. There are considerable doubts regarding the effectiveness of this screening (Stirling *et al*, 2005), which may explain the high rate of BRRO. This screening is not available in the UK outside research trials.

Men did not report significantly different levels of general distress at baseline or 3-year follow-up, which suggests that testing for BRCA1/2 genetic mutations does not adversely affect men's mental health. This study provides a snapshot of screening undergone by men following BRCA1/2 testing. Although a relatively small group, there were significant differences between carriers and non-carriers in levels of colorectal and prostate screening. There are no guidelines for the risk management of men with BRCA1/2 mutations although research is underway (IMPACT: targeted screening for prostate cancer). Since the study commenced bowel cancer risks have become less clear, especially for BRCA1. Colorectal screening in gene carriers is not generally recommended unless there is also a significant family history of colorectal cancer. From April 2006 a national screening programme for colorectal cancer was implemented across the UK (Department of Health, 2006).

Rates of risk reducing surgery varied by clinical genetics centre and suggest differences in clinical practice impact on risk management options. Research in larger populations having risk reducing surgery is warranted. It would be important, now that guidelines for risk management are in place, to collect further data on standardisation between centres and equity of access to risk management services within the NHS.

A substantial number of female carriers (40%) reported difficulties with insurance post-genetic test. In the year following predictive testing 20% of female carriers reported problems with insurance (Watson *et al*, 2004). This indicates that problems with insurance persist in the long term. We set out to assess the level of problems experienced around insurance. The high rate of problems warrants further investigation as precise details of difficulties experienced were not provided by the women in this study. Further work is needed to explore the precise nature of problems experienced. This is a sensitive area and an in-depth qualitative approach is likely to be appropriate in future research.

In summary, many female carriers opt for risk reducing surgery post-genetic test and engage in screening, although this varies across genetics centres. A substantial number of non-carriers ⩾50 did not have a mammogram in the 3 years post-genetic test contrary to National Breast Screening recommendations. Some non-carriers may be deriving false reassurance from the genetic test result. If this is confirmed in other cohorts, there may be some education needed for women who do not have a genetic fault. In addition, our data suggest that support may be needed for some female non-carriers in the long term who report concerns about developing cancer similar to pre-genetic test levels where an initial reduction in worry was reported. We have limited data regarding insurance difficulties experienced by women post-genetic test result but genetic testing does seem to be raising problems.

## Figures and Tables

**Table 1:** Socio-demographic characteristics of the participants

	**Female**	**Male**	**Total**
Participants	154	39	193
			
*Genetic status*			
Carriers	53	18	71
Non-carriers	101	21	122
			
*Age (years)* a			
Median (Range)	42 (23–72)	52 (28–86)	43 (23–86)
<35	33	5	38
35–49	81	12	93
⩾50	39	22	51
			
*N with offspring*^a^ *(daughters)*			
No. of offspring	21	3	24
1 or more	132 (104)	36 (29)	168 (133)
Unknown	1	0	1

Figures shown are *N*-values unless indicated otherwise.

a At baseline.

**Table 2:** Risk management options given as percentages undertaken by women at baseline and in the 3 years post-testing according to carrier status and age

		**3 year follow-up**	
	**Baseline**	***N*=154**	
**Age group**	***N*=227**	**Carrier (*n*=53)**	**Non-carrier (*n*=101)**
*Mammography*			
<35	29	81	22
35–49	46	91	44
>50	61	100	65
Total	45	89	46
			
BRRM*			
<35	2	38	0
35–49	2	38	0
⩾50	0	0	0
Total	1	34	0
			
*BRRO+*			
<35	2	25	0
35–49	10	54	3
>50	30	40	7
Total	13	43	3
			
*Breast biopsy* ^*^			
<35	Not asked	19	9
35–49		19	2
⩾50		20	6
Total		19	5
			
*Ovarian biopsy+*			
<35	Not asked	13	0
35–49		4	0
>50		0	4
Total		6	1
			
*Ovarian US+*			
<35	8	81	5
35–49	32	75	16
⩾50	26	60	25
Total	43	75	17
			
*CBE*			
<35	35	88	39
35–49	48	81	51
⩾50	48	100	38
Total	45	85	44
			
*Tamoxifen*			
<35	0	0	0
35–49	2	6	0
⩾50	10	20	6
Total	3	6	2
			
*BrSE*			
<35	85	81	100
35–49	91	97	98
⩾50	83	100	88
Total	88	91	95

Figures exclude patients who have had BRRM (^*^) or BRRO (+) as appropriate.

**Table 3:** General mental health and cancer specific worry

	**Baseline**		**3-year follow-up**		
	**Carriers**	**Non-carriers**	**Carriers**	**Non-carriers**	***P*** *
*GHQ females*					
Mean (s.d)	2.7 (4.6)	2.6 (3.8)	4.5 (6.3)^a^	3.7 (5.3)	0.3
Median (range)	0 (0–20)	1 (0–15)	1 (0–27)	1 (0–24)	
					
*GHQ males*					
Mean (s.d)	0.8 (2.6)	1.9 (3.5)	1.6 (3.6)	2.5 (5.1)	0.8
Median (range)	0 (0–11)	0 (0–12)	0 (0–12)	0 (0–17)	
					
*GHQ case (⩾10)*					
*n* female	6 (12%)	8 (8%)	9 (18%)	16 (17%)	1.0
*n* male	1 (6%)	2 (10%)	2 (11%)	2 (10%)	
					
*CWS-R females*					
Mean (s.d)	11.7 (3.1)	11.5 (3.4)	10.4(3.6)^a^	9.3 (2.1)	0.2
Median (range)	11 (6–21)	11 (6–22)	10 (6–21)	9 (6–15)	
					
*CWS-R males*					
Mean (s.d)	Not measured		8.2 (2.0)	8.1 (2.0)	
Median (range)			8 (6–13)	8 (6–11)	

GHQ with binary scoring and ⩾10 cutoff to indicate cases.

* Comparison of the change from baseline of carriers *vs* non-carriers.

a Significant change from baseline *P*=0.03.
